# Identification of predominant genes involved in regulation and execution of senescence-associated nitrogen remobilization in flag leaves of field grown barley

**DOI:** 10.1093/jxb/eru094

**Published:** 2014-04-03

**Authors:** Julien Hollmann, Per L. Gregersen, Karin Krupinska

**Affiliations:** ^1^Institute of Botany, Christian-Albrechts-University of Kiel, Olshausenstraße 40, 24098 Kiel, Germany; ^2^Department of Molecular Biology and Genetics, Aarhus University, AU-Flakkebjerg, DK-4200 Slagelse, Denmark

**Keywords:** Barley, field experiment, flag leaf, *Hordeum vulgare* L., nitrogen remobilization, nitrogen supply, leaf senescence, HvNAC026.

## Abstract

The transcriptomes of senescing flag leaves collected from barley field plots with standard or high nitrogen supply were compared to identify genes specifically associated with nitrogen remobilization during leaf senescence under agronomically relevant conditions. In flag leaves collected in field plots with high nitrogen supply, the decline in chlorophyll content was delayed. By comparing changes in gene expression for the two nitrogen levels, it was possible to discriminate genes related to nitrogen remobilization during senescence and genes involved in other processes associated with the late development of leaves under field conditions. Predominant genes that were more strongly upregulated during senescence of flag leaves from plants with standard nitrogen supply included genes encoding the transcription factor HvNAC026, serine type protease SCPL51, and the autophagy factors APG7 and ATG18F. Elevated expression of these genes in senescing leaves from plants with standard nitrogen supply indicates important roles of the corresponding proteins in nitrogen remobilization. In comparison, the genes upregulated in both flag leaf samples might have roles in general senescence processes associated with late leaf development. Among these genes were the transcription factor genes *HvNAC001, HvNAC005, HvNAC013, HvWRKY12* and MYB, genes encoding the papain-like cysteine peptidases HvPAP14 and HvPAP20, as well as a subtilase gene.

## Summary

Barley genes potentially involved in nitrogen remobilization during leaf senescence under field conditions were identified by microarray analyses.

## Introduction

Leaf senescence is a highly regulated complex developmental process terminated by death of the organ. The purpose of senescence is to remobilize nutrients, in particular nitrogen, from the senescing organs to still growing plant parts, and finally to the developing seeds. In crop plants such as cereals, senescence is a slow process, whereas in other plants, in particular *Arabidopsis*, senescence might occur quickly, and hence has been discussed as a cell-death process (Jansson and Thomas, 2008).

In crop plants, leaf senescence is of pivotal importance for yield (Gregersen *et al.*, 2013). It has been estimated that during seed filling of most annual crop plants more than 70% of the leaf nitrogen is exported from the senescing leaves (Peoples and Dalling, 1988). Chloroplast proteins are the major source for grain nitrogen (Hörtensteiner and Feller, 2002). Up to 70% of the chloroplast nitrogen is assembled into ribulose-1,5-bisphosphate carboxylase/oxygenase (Rubisco). Another major fraction is contained in the chlorophyll-protein complexes of the thylakoid membrane. As long as chlorophyll is bound to these proteins, they are not accessible to proteases (Hörtensteiner and Feller, 2002). The decline in chlorophyll content preceding the degradation of the membrane proteins is therefore an excellent marker of leaf senescence (Humbeck *et al.*, 1996).

Senescence can be accelerated under situations of low nitrogen supply (Schulze *et al.*, 1994), or it can be delayed or even reversed by excess nitrogen supply (Diaz *et al.*, 2005; Schildhauer *et al.*, 2008). A low nitrogen nutrition status during senescence is also known to promote proteolysis (Masclaux *et al.*, 2000). Despite their importance for plant productivity, knowledge of the genes encoding the major proteases involved in senescence-associated degradation of chloroplast proteins and the factors regulating protein breakdown remains scarce. Based on gene expression studies, several proteases that are encoded by genes upregulated during senescence were identified, but their substrates are not known (Bhalerao *et al.*, 2003; Gepstein *et al.*, 2003; Guo *et al.*, 2004a; Buchanan-Wollaston *et al.*, 2005; Gregersen and Holm, 2006).

The intention of this study was to identify genes in barley encoding proteins that might be important for nitrogen remobilization during leaf senescence under agronomically relevant conditions in the field. For this purpose the transcriptomes of flag leaves collected from field plots supplied with two different levels of nitrogen were analysed. Flag leaves have been chosen because their senescence is known to be most important for seed filling (Stoy, 1973; Stamp and Herzog, 1976). Senescence symptoms in flag leaves of plants grown at high nitrogen supply were suppressed compared with flag leaves from plots with standard nitrogen supply. To identify key factors involved in regulation of nitrogen remobilization the first goal of this microarray analysis was to identify genes encoding transcription factors. The second goal was to identify genes encoding enzymes putatively involved in protein degradation.

## Materials and methods

### Plant material

The winter barley cultivar Lomerit (*Hordeum vulgare* L., cv. Lomerit), introduced in 2001, has been one of the most cultivated winter barley varieties in Germany for several years. The field trials were conducted at the Experimental Station Hohenschulen belonging to the Agricultural Faculty of the University of Kiel (see also Krupinska *et al.*, 2012). Plants were grown with a density of 300 plants m^–2^ in plots of 3×8 m. The plots were distributed within a large field. The field used in 2010 was adjacent to the field used in 2009. For both fields, rapeseed, wheat, and oat were used as previous crops. During crop rotation rapeseed and oat were consistently fertilized, wheat and barley plots were inversely fertilized. Thus, the nitrogen content of the soil before application of fertilizer was probably similar in both years.

Barley field plots fertilized with a total standard nitrogen supply of 80kg per hectare were designated as standard nitrogen plots (SN) and those fertilized with a total nitrogen supply of 240kg per hectare were designated as plots with high nitrogen supply (HN). Flag leaves from four plots each of SN and HN were collected between 9.00 and 11:30 CET (Central European Time) from 27 May to 20 June in 2009, and from 12 June to 4 July in 2010. Meteorological data on air temperature (107 Temperature Probe, Campbell Scientific, Bremen, Germany), rainfall (Ombrometer, Thies Clima, Göttingen, Germany) and global radiation (SP-Lite, Thies Clima, Göttingen, Germany) were acquired from devices installed next to the field (Krupinska *et al.*, 2012).

### Determination of chlorophyll contents

The relative chlorophyll content of flag leaves was determined in the field non-destructively using a Minolta SPAD-502 (Konica Minolta Sensing, Osaka, Japan). The measurements were done 2cm above the bases of the leaves (20 leaves in each plot, four plots in total).

### Determination of the ear dry weight

To determine the average dry weights of the ears, ears were collected at each time of leaf sampling (four plants in each plot, four plots in total) and were dried until their weights were constant.

### Determination of the leaf protein content

For protein determination eight flag leaves (two leaves from each plot, four plots in total) were frozen and powdered in liquid nitrogen. Before freezing, leaf area was determined using SigmaScan (Systat, San Jóse, California, USA). Proteins were extracted as previously described (Pötter and Kloppstech, 1993). The protein content was determined using the Bio-Rad Protein Assay (Bio-Rad Laboratories GmbH, Munich, Germany) according to manufacturer’s instructions.

### Isolation of RNA

Samples were frozen and powdered in liquid nitrogen, total RNA was extracted using the Spectrum Plant Total RNA Kit (Sigma, Steinheim, Germany) according to manufacturer’s instructions. RNA concentrations were determined by using a NanoDrop ND-1000 (NanoDrop Technologies, Inc., Wilmington, Delaware, USA), quality was verified by electrophoresis on 1% agarose gels. RNA samples used for the microarray experiment were additionally verified by using a Bioanalyzer 2100 (Agilent Technologies, Inc., Santa Clara, USA) conducted by ImaGenes (Berlin, Germany).

### Microarray analysis

Microarray analyses were performed using three replicate samples of flag leaves collected from SN as well as HN field plots in 2009. Preparation and hybridizations of the samples to the 4x44k barley gene expression microarrays (Agilent Technologies, Inc., Santa Clara, USA) were conducted by ImaGenes (Berlin, Germany).

For data analysis, the limma package version 2.2.0 (Smyth, 2005) of the R software version 2.2.0 (R development core team, 2005) was used. Raw data sets were corrected for background signals using the limma function “half”. Normalization between arrays was done by using the ‘quantile’ function (Bolstad *et al.*, 2003). Differentially expressed genes were identified using the ‘lmFit’ and the ‘contrast.fit’ functions of the limma package. The Blast algorithm was used to screen the microarray dataset as well as publicly available databases. Redundancies of the probes of the 44k microarray were eliminated by a BlastN search of the barley genome assembly (Mayer *et al.*, 2011) and the HarvEST 35 assembly, both available at HarvEST (http://www.harvest-web.org, last accessed 24 February 2014), as well as by a BlastN search of a recently generated transcriptome assembly (Kohl *et al.*, 2012). Initially, functional classification and annotation of genes was performed using the Mercator software (Usadel *et al.*, 2009). Results were subsequently checked by BlastX searches of the non-redundant database from NCBI (http://www.ncbi.nlm.nih.gov, last accessed 24 February 2014) and the MEROPS peptidase database (Rawlings *et al.*, 2012). To generate heat maps, the Cluster 3.0 Software and TreeViewer 1.1.3 were used. For simplicity, sequences included on the microarray or in the qRT-PCR were designated in this work as genes.

### Quantitative real-time PCR

**Fig. 3.**
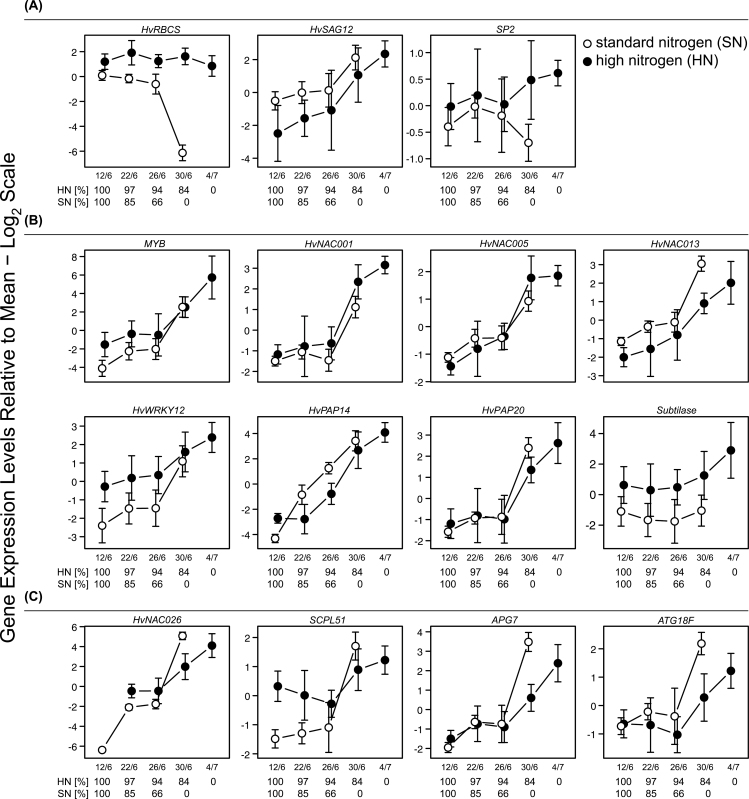
Relative expression levels of selected genes as analysed by quantitative real-time PCR. RNA samples were prepared from leaves of plants with standard nitrogen supply (SN) and high nitrogen supply (HN), respectively. Leaves were collected every fourth day from 12 June (12 days after flowering of SN and HN plants) until 4 July 2010. The relative chlorophyll content at 12 June was set to 100% (Supplementary Fig. S1A, available at *JXB* online). The expression data are scaled relative to the overall mean, error bars indicate SD and are derived from three biological replicates, *n*=3. (A) Controls and senescence marker, (B) genes showing higher expression in HN than in SN samples, and (C) genes showing higher expression in SN than in HN samples.

RNA samples from leaves collected in 2010 were used for cDNA synthesis using the SuperScript II Reverse Transcriptase Kit (Invitrogen, Carlsbad, CA, USA) according to manufacturer′s instructions. All reactions were done in three biological replicates, and for each of the biological replicates three technical replicates were measured. Primer design and quantitative real-time PCR were performed as described previously (Christiansen *et al.*, 2011) using the ABI Prism7900HT Sequence Detection System and the Power SYBR Green PCR master Mix (Applied Biosystems, Foster City, USA). As references the gene encoding the 18S ribosomal RNA (*AK251731*) and the gene encoding the splicing factor 2 (SP2) (*AK249101*) (Fig. 3A) were used. The primer sequences are listed in Supplementary Table S2 (available at *JXB* online).

Ct values and fold changes were calculated by the R software (R developmental core team, 2005) using the HTqPCR package (Dvinge and Bertone, 2009).

## Results

### Senescence of flag leaves in the field at standard nitrogen supply

**Fig. 1.**
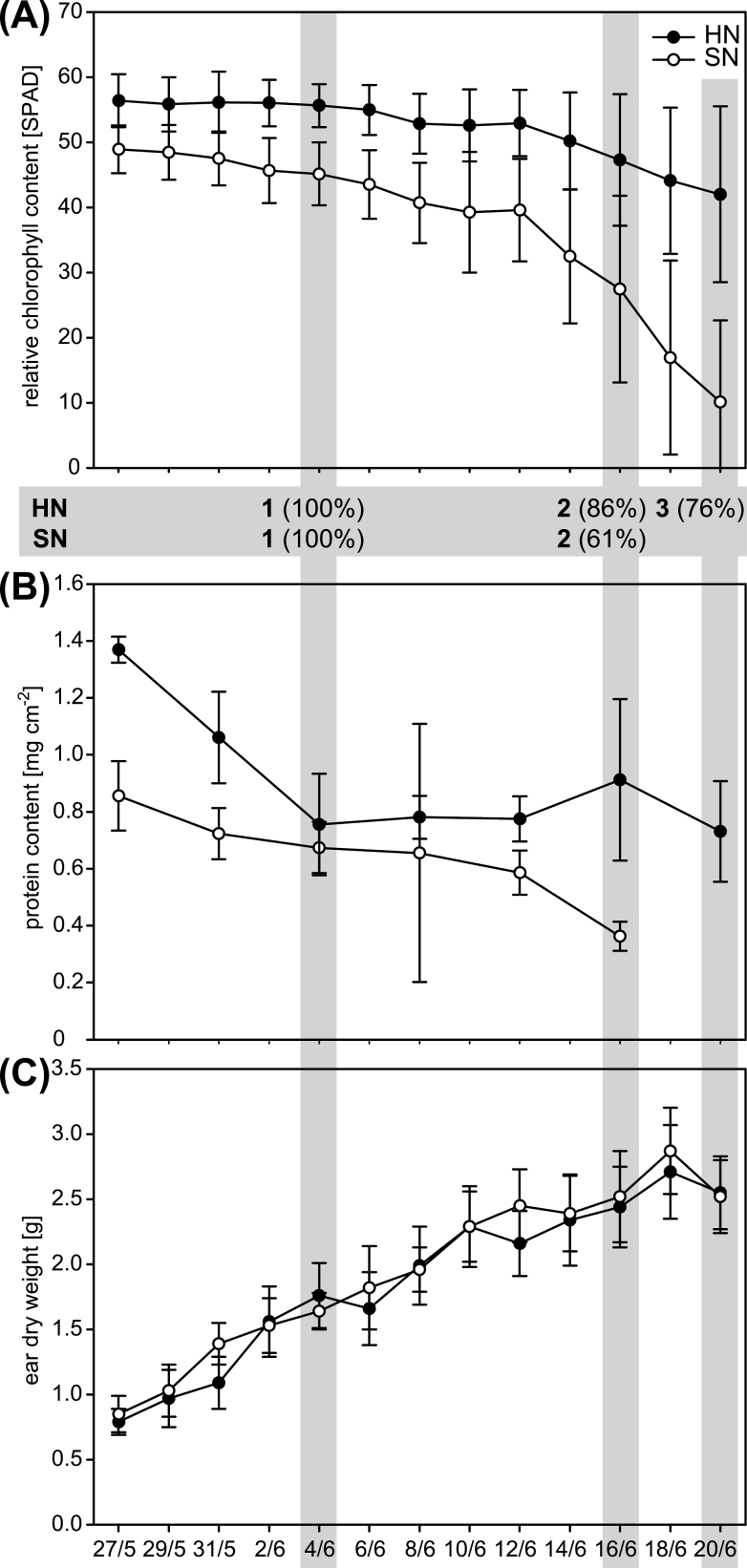
Characterization of flag leaves and ears collected from barley plants grown in field plots supplied with standard (SN) or high nitrogen (HN) in the period from 27 May (16 days after flowering of SN and HN plants) until 20 June 2009. Samples used for microarray analyses are highlighted in grey. (A) Relative chlorophyll content. Error bars indicate standard deviations (SD), *n*=80. (B) Protein content of leaves. Error bars indicate SD, *n*=8. (C) Dry weight of the ears. Error bars indicate SD, *n*=16.

For characterization of senescence of flag leaves from the 2009 field plots, the relative chlorophyll contents and protein contents were determined (Fig. 1A, B). Although the relative chlorophyll content of leaves from standard-nitrogen plots (SN) in the sampling period from 4 until 20 June 2009 declined by 80%, the chlorophyll content of leaves from high-nitrogen plots (HN) declined by only 24% during the same period. By 12 June 2009, the decrease in chlorophyll content was accelerated in leaves collected from plots of both nitrogen supplies. This date was defined as the day of senescence onset (Krupinska *et al.*, 2012). The protein content decreased in SN leaves during the whole sampling period until 16 June 2009 to a level of 42% compared with the initial protein content. In HN leaves the protein content decreased from 27 May 2009 until 4 June 2009 to a level of 55% compared with the initial protein content, and stayed almost constant thereafter (Fig. 1B). Grain filling was monitored by ear dry weight, which increased continuously during the sampling period and reached a maximum at 18 June 2009 (Fig. 1C). No significant differences were observed between the dry weight of SN ears and HN ears indicating that the standard nitrogen supply did not limit the filling of the kernels.

In comparison to 2009 the development of the plants in 2010 was delayed by about 20 days (days of flowering were 11 May 2009 and 31 May 2010). This was compensated partly by a shorter period between the heading date and the onset of senescence (Fig. 1; Supplementary Fig. S1, available at *JXB* online).

### Gene expression analyses by microarrays

To identify the predominant genes important for nitrogen-remobilization processes, senescing flag leaves from SN field plots were compared with flag leaves collected at the same time from HN field plots. Changes in gene expression during senescence were analysed using the 4x44k barley microarray. Raw datasets generated by scanning of the hybridized arrays are available on ArrayExpress (E-MTAB-2202).

**Fig. 2.**
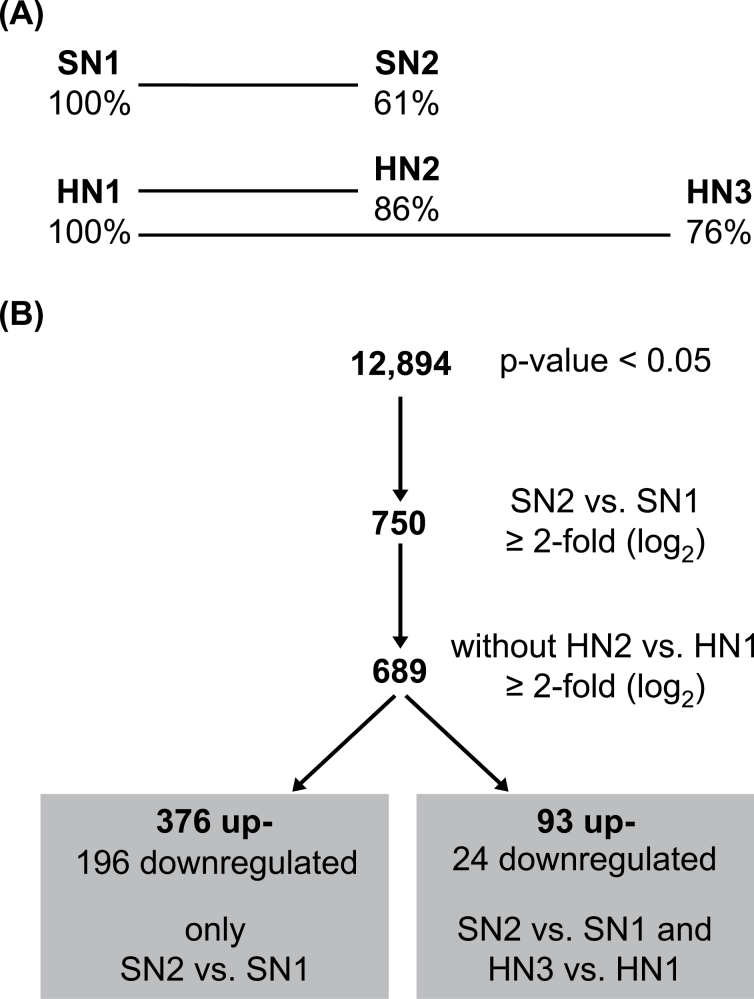
(A) Microarray design used to analyse differences between non-senescing and senescing samples from plants grown under standard nitrogen (SN) or high nitrogen (HN) supply. Relative chlorophyll contents (Fig. 1A) are given in percentages. Each line represents one comparison. (B) Filtering of the microarray results. Conditions for each filtering step are given. The procedure resulted in two groups of genes: genes only differentially expressed in the comparison SN2 vs. SN1 and genes differentially expressed in both comparisons SN2 vs. SN1 and HN3 vs. HN1.

The leaves collected on 4 June 2009 from SN and HN plots (SN1, HN1) were chosen as non-senescent control leaves. Expression changes between senescing SN leaves and SN control leaves that were not senescing (SN2 vs. SN1) were compared with the changes in gene expression occurring in the same period of time in HN leaves (HN2 vs. HN1) (Fig. 2A). At the second sampling time point (SN2, HN2) the chlorophyll content in SN flag leaves was reduced by 39%, whereas in HN leaves it was reduced by only 14%. Importantly, both samples were collected at the same time of the day. It is expected that changes in gene expression caused by environmental factors such as light and temperature occur simultaneously in both samples and are eliminated by the subtraction of the datasets (Fig. 2B). To distinguish between genes involved in nitrogen remobilization and other developmentally regulated genes, expression changes during senescence of SN leaves (SN2 vs. SN1) were compared with changes in gene expression in the oldest HN leaves (HN3 vs. HN1), which were collected at 20 June 2009 (Fig. 2B). At this third sampling time point (HN3) the chlorophyll content in HN3 flag leaves was reduced by only 24% compared with the first time point (HN1).

Reduction of redundancies of the probes on the 44k barley microarray and statistical analysis in limma resulted in 12,894 genes that appeared to be unique and had an adjusted *P*-value smaller than 0.05 in the combined analysis for differentially expressed genes (Fig. 2B). Of the differentially expressed genes, 750 showed at least 2-fold (log_2_) changes in expression in the comparison of SN2 vs. SN1 (Fig. 2B; Supplementary Table S1, available at *JXB* online) and these were chosen for further analysis. To eliminate expression changes due to environmental factors, the 61 genes, which were also differentially expressed during the same period in HN leaves (HN2 vs. HN1), were subtracted from this gene pool (Fig. 2B; Supplementary Table S1, available at *JXB* online).

Among the remaining 689 genes, 117 (93 up- and 24 downregulated) were also differentially expressed during further development of HN leaves (HN3 vs. HN1) and 572 (376 up- and 196 downregulated) genes were differentially expressed in senescing SN leaves (SN2 vs. SN1) (Fig. 2B; Supplementary Table S1, available at *JXB* online). 75 of the 117 genes differentially expressed in both SN and HN leaves (SN2 vs. SN1 and HN3 vs. HN1) as well as 330 of the 572 genes with altered expression levels in SN leaves (only in SN2 vs. SN1) could be assigned with high accuracy to a total of 27 functional categories called BIN codes (Thimm *et al.*, 2004) (Supplementary Fig. S2; Supplementary Table S1, available at *JXB* online). Overall, the genes identified as differentially expressed belong mainly to the categories transport, development, signalling, protein, and RNA (Supplementary Table S1, available at *JXB* online). With regard to nitrogen remobilization, the analysis was focused on genes involved in degradation and regulation processes (Table 1).

**Table 1.** Fold changes in expression as determined by the microarray hybridization of genes assigned to the categories ‘RNA/regulation of transcription’ and ‘protein/degradation’ (A) Genes upregulated (min. 2-fold log_2_) in both senescing flag leaves of plants from plots with standard (SN2 vs. SN1) and excess (HN3 vs. HN1) nitrogen supply. (B) Genes exclusively upregulated (min. 2-fold log_2_) in senescing flag leaves of plants from plots with standard nitrogen supply (SN2 vs. SN1). If possible, gene names are indicated for either barley or for the orthologous genes in *Arabidopsis*. Genes tested by qRT-PCR are marked by asterisks.

Accession	Name	BIN Description	SN	HN		SN vs. HN		
			2 vs. 1	2 vs. 1	3 vs. 1	1 vs. 1	2 vs. 2	2 vs. 3
**(A) protein.degradation**								
CA019173			2,60	1,93	3,32	2,02	2,69	1,30
TA40019_4513	ATG8	autophagy	2,82	1,27	2,16	0,63	2,18	1,30
AM941123*	HvSAG12	cysteine protease	2,59	1,38	2,18	0,92	2,13	1,33
AM941124*	HvPAP14	cysteine protease	5,26	1,17	2,92	0,55	4,64	2,89
BF256720	RD21	cysteine protease	2,18	1,06	2,17	0,35	1,47	0,35
TA42918_4513*	Subtilase	subtilases	6,13	1,91	4,03	0,43	4,66	2,53
TA36132_4513	UBC5	ubiquitin.E2	3,76	1,28	2,71	1,18	3,66	2,23
BG414942		ubiquitin.E3.RING	3,65	1,28	2,46	0,72	3,09	1,92
TA46919_4513		ubiquitin.E3.RING	3,57	1,53	2,20	0,67	2,72	2,04
CA008354		ubiquitin.E3.RING	2,99	1,28	2,10	0,87	2,58	1,76
TA37012_4513	RGLG2	ubiquitin.E3.RING	2,22	1,03	2,04	0,17	1,36	0,34
TA35901_4513		ubiquitin.E3.SCF.FBOX	3,84	1,58	2,57	0,57	2,82	1,84
TA35023_4513	UBP5	ubiquitin.ubiquitin protease	2,61	0,65	2,78	-0,13	1,83	-0,30
**RNA.regulation of transcription**								
TA48008_4513*	MYB	MYB-related transcription factor family	3,91	0,77	2,10	0,27	3,42	2,08
TC163636*	HvNAC013	NAC domain transcription factor family	3,32	1,45	2,90	0,77	2,64	1,18
AK251058*	HvNAC005	NAC domain transcription factor family	2,15	1,88	2,34	1,15	1,43	0,96
TC164038		putative transcription regulator	3,48	1,02	2,32	0,98	3,45	2,14
BM370440*	HvWRKY12	WRKY domain transcription factor family	3,24	1,64	2,72	0,40	2,00	0,91
**(B) protein.degradation**								
BF258397			2,60	0,73	1,41	0,35	2,22	1,53
BU985502	AAH-2		2,16	0,58	1,17	0,49	2,07	1,48
TA38933_4513		AAA type	2,34	0,39	0,52	0,21	2,16	2,03
TA52242_4513*	APG7	autophagy	3,80	-0,66	0,50	-1,06	3,40	2,23
BF622783	ATG3	autophagy	3,16	0,59	1,66	0,43	3,00	1,93
BE216622	APG8A	autophagy	3,12	0,94	1,99	0,58	2,77	1,72
BQ471466		autophagy	2,56	0,61	1,25	0,58	2,53	1,88
AM085509	APG9	autophagy	2,40	0,64	1,43	0,42	2,19	1,39
TA56628_4513*	ATG18F	autophagy	2,35	0,44	1,19	0,92	2,83	2,08
BF628472	ATG8C	autophagy	2,13	0,86	1,08	0,18	1,45	1,23
AM941127*	HvPAP20	cysteine protease	4,27	0,84	1,69	-0,10	3,32	2,48
AM941122	XBCP3	cysteine protease	3,66	1,14	1,98	-0,02	2,50	1,65
AM941116		cysteine protease	2,37	0,62	1,01	0,42	2,18	1,79
TA45222_4513		cysteine protease	2,36	0,44	0,97	0,31	2,23	1,71
EX594583		cysteine protease	2,24	0,78	1,38	0,52	1,98	1,38
CD663018	RD19	cysteine protease	2,17	0,72	1,32	0,38	1,82	1,22
AK251038*	SCPL51	serine protease	3,56	1,14	1,63	0,30	2,72	2,23
TA57593_4513	ATRBL1	serine protease	2,61	0,15	0,48	-0,13	2,33	1,99
BY868089	UBC11	ubiquitin.E2	2,24	0,66	0,96	0,32	1,91	1,61
TA53580_4513		ubiquitin.E3.BTB/POZ Cullin3.BTB/POZ	3,93	0,10	1,64	0,50	4,33	2,79
BY868672		ubiquitin.E3.BTB/POZ Cullin3.BTB/POZ	2,43	0,49	0,86	0,07	2,01	1,63
TA44869_4513		ubiquitin.E3.RING	2,92	1,28	1,60	0,22	1,86	1,54
CA008421		ubiquitin.E3.RING	2,35	0,72	1,49	0,38	2,01	1,25
BM816188		ubiquitin.E3.RING	2,13	0,56	1,11	0,38	1,95	1,40
TA34605_4513		ubiquitin.E3.SCF.FBOX	2,80	0,70	1,14	0,51	2,61	2,17
AK248328		ubiquitin.E3.SCF.FBOX	2,25	0,42	1,16	0,30	2,13	1,39
CX632454		ubiquitin.E3.SCF.FBOX	2,10	0,38	1,01	-0,30	1,41	0,79
TA31497_4513		ubiquitin.proteasome	2,03	0,76	1,31	0,22	1,49	0,93
TA57316_4513	UBQ3	ubiquitin.ubiquitin	2,72	1,45	1,55	0,14	1,41	1,31
**RNA.regulation of transcription**								
BE421551	IAA16	Aux/IAA family	3,22	0,04	0,84	0,56	3,74	2,94
EX590103		C2C2(Zn) CO-like	2,03	0,41	0,90	0,11	1,73	1,25
AK250112	AFO	C2C2(Zn) YABBY family	2,42	0,37	0,65	0,33	2,38	2,10
DQ151543		C2H2 zinc finger family	2,17	0,42	1,32	-0,03	1,72	0,82
TC164854*	HvNAC026	NAC domain transcription factor family	4,20	0,32	1,02	-0,05	3,83	3,14
EX573211*	HvNAC001	NAC domain transcription factor family	2,22	1,09	-1,30	0,17	1,30	1,19
TA39240_4513	HvNAC	NAC domain transcription factor family	3,06	1,45	1,28	0,20	1,81	1,98
CK124370		putative transcription regulator	3,01	0,41	1,20	0,47	3,07	2,29
TA39614_4513		putative transcription regulator	2,71	1,11	1,70	0,25	1,86	1,27
BJ448756		putative transcription regulator	2,51	0,48	0,72	0,48	2,51	2,28
TA43937_4513		unclassified	2,49	-0,15	0,21	-0,56	2,08	1,72
TA43565_4513		unclassified	2,38	0,81	1,83	0,53	2,11	1,08
EX578789	ATG18D	unclassified	2,34	0,64	1,25	0,03	1,74	1,12

The 93 genes upregulated at late developmental stages from both SN (SN2 vs. SN1) and HN (HN3 vs. HN1) plots might play roles in age-dependent processes occurring independent of nitrogen supply. Among these 93 genes, five genes were identified encoding transcription factors and 13 genes encoding proteins putatively involved in processes associated with protein degradation (Table 1A). The transcription factors included MYB, HvNAC013, HvNAC005 and HvWRKY12. Among the genes associated with protein degradation, four encoded peptidases including HvSAG12, seven encoded factors of the ubiquitin-proteasome-system and one was the barley orthologue of the *Arabidopsis* autophagy-related gene *ATG8*. *HvSAG12* and one of its orthologues in *Arabidopsis*, encode a cysteine protease which is used as a senescence marker gene (Lohman *et al.*, 1994). Its senescence-associated upregulation was previously shown in barley (Jukanti *et al.*, 2008; Parrott *et al.*, 2010) and wheat (Gregersen and Holm, 2006; Ruuska *et al.*, 2008).

Leaves of plants with standard nitrogen supply (SN) showed typical symptoms of senescence. The genes strongly upregulated in these leaves (SN2 vs. SN1) hence might play roles in proteolysis and nitrogen remobilization. The two genes with highest upregulation during senescence of SN leaves were *HvPAP20*, encoding a cysteine peptidase, and *HvNAC026*, encoding a NAC transcription factor (Table 1B). Furthermore, 13 genes encoding putative transcription factors and 29 genes putatively involved in protein degradation processes were identified. Most of the transcription factors can be assigned to the NAC, C2C2(Zn), C2H2, and IAA families. The other genes encode eight peptidases, 11 factors of the ubiquitin-proteasome-system (UPS), seven autophagy-related factors, and three other proteins putatively involved in protein degradation.

### Quantitative analyses of the expression of selected genes

Microarray analysis showed that 376 genes were highly upregulated mainly in senescing SN leaves (SN2), whereas 93 genes were upregulated in both senescing SN (SN2 vs. SN1) and HN (HN3 vs. HN1) flag leaves. To investigate whether the differences in expression levels of genes selected by microarray hybridizations are indeed caused by different quantities of nitrogen supply in the field, barley plants were grown in the following year (2010) in field plots supplied with the same two quantities of nitrogen. Again, flag leaves were collected from SN and HN plots. Expression of selected genes was analysed by quantitative real-time PCR (qRT-PCR) (Table 1, marked by asterisk; Fig. 3B, C).

qRT-PCR was done with RNA from flag leaves collected in 2010 at five stages of development. These stages were characterised by the relative chlorophyll (Supplementary Fig. S1A, available at *JXB* online) and protein contents (Supplementary Fig. S1B, available at *JXB* online) of the leaves. In addition, ear dry weight was determined (Supplementary Fig. S1C, available at *JXB* online). The relative chlorophyll content in HN and SN flag leaves at 12 June was set to 100%. Typically for senescing leaves, the transcript level of the gene coding for the small subunit of Rubisco (HvRBCS) declined in SN leaves during the sampling period, whereas in HN flag leaves its level was rather stable (Fig. 3A). Inversely, the transcript level of the senescence marker gene *HvSAG12* (Jukanti *et al.*, 2008; Parrott *et al.*, 2010) increased during development in both SN and HN leaves (Fig. 3A). Genes showing senescence-associated upregulation in both SN and HN leaves such as the *SAG12* gene (Fig. 3A) might have functions in other senescence-associated processes than in nitrogen remobilization. Additional genes, which showed upregulation during leaf development independent of the nitrogen supply, encode the transcriptions factors MYB, HvNAC001, HvNAC005, HvNAC013, and HvWRKY12, as well as the two papain-like cysteine peptidases HvPAP14 and HvPAP20, and a gene encoding a subtilase (Fig. 3B). Among the genes showing highly increased expression during senescence of SN leaves were the genes encoding the transcription factor HvNAC026, a gene encoding an orthologue of the serine protease SCPL51 in *Arabidopsis* as well as two genes homologous to the genes encoding autophagy-associated proteins ATG7 and ATG18F in *Arabidopsis* (Fig. 3C).

Most of the genes selected by microarray hybridizations (Table 1) showed comparable tendencies in the expression profiles by qRT-PCR analysis (Fig. 3). Exceptions were the genes *HvNAC001* and *HvPAP20*, which in 2010 showed again a strong upregulation in senescing SN leaves but also in the elder HN leaves. The expression of the subtilase gene in SN leaves differed in 2010 from the previous year.

## Discussion

Characterization of senescence of flag leaves collected from field-grown barley plants and determination of kernel weight during the time of leaf sampling showed that the standard nitrogen supply provided in this study did not limit the filling of the kernels (Fig. 1; Supplementary Fig. S1, available at *JXB* online). The high nitrogen supply in HN plots was in excess and hence could diminish/suppress the development of senescence symptoms such as chlorophyll degradation. This situation allowed identification of genes putatively involved in protein degradation and regulation processes important under nitrogen-limiting conditions using the pool of genes upregulated mainly in SN (SN2 vs. SN1) flag leaves.

For this study flag leaves were chosen, because their senescence is of pivotal importance for grain filling (Stoy, 1973; Stamp and Herzog, 1976). The most prominent changes in gene expression were detected in the SN treatment (SN2 vs. SN1). To identify genes specifically involved in nitrogen remobilization during senescence occurring at standard nitrogen supply (SN), genes upregulated during development of flag leaves independent of the nitrogen supply (HN) were excluded from this gene pool. The number of genes relevant for nitrogen remobilization was furthermore reduced by a very stringent minimum fold change of 2 (log_2_). By this approach 13 genes encoding transcription factors, and 29 genes encoding proteins putatively involved in protein degradation processes were identified as most strongly upregulated in senescing SN leaves (Table 1B). A qRT-PCR with similar samples collected in 2010 further reduced the number of relevant genes. Among the genes selected by microarray hybridization only the genes encoding the transcription factor HvNAC26 as well as the genes *SCPL51*, *APG7*, and *ATG18F* were reproducibly upregulated specifically in leaves undergoing nitrogen remobilization. As these analyses were done on material grown in the fields and in addition in two consecutive years several environmental factors could influence the development and behaviour of the plants. Thus, the weather conditions in 2010 (Krupinska *et al.*, 2012) led to a delayed flowering time point and shortened time between flowering and senescence (Fig. 1A; Supplementary Fig. S1, available at *JXB* online). However, by using two adjacent fields cultivated in the same crop rotation and fertilized with equal amounts for several years, differences of the initial nitrogen amount in the soil were reduced as much as possible.

### Transcription factors

In senescent leaves from plots with standard (SN2 vs. SN1) or excess (HN3 vs. HN1) nitrogen supply, genes encoding transcription factors could be identified that might play roles in age-related processes other than leaf nitrogen remobilization. This group of genes included genes encoding a MYB transcription factor, two NAC transcription factors (HvNAC013, HvNAC005) and the transcription factor HvWRKY12 (Table 1A). By qRT-PCR with samples collected in 2010 these results could be confirmed (Fig. 3B). Expression of the gene encoding the MYB factor was also shown to be upregulated in germinating seeds (Zhang *et al.*, 2004), as well as in developing seeds 14–21 days past anthesis (Jukanti and Fischer, 2008). The *HvNAC013* gene was shown to be elevated in response to abscisic acid (ABA) and methyl jasmonate (MeJA) (Christiansen *et al.*, 2011). The HvNAC013 protein was shown to interact with the barley RADICAL-INDUCED CELL DEATH 1 protein (RCD1) (Kjaersgaard *et al.*, 2011), which is involved in regulation of genes responsive to stress and regulated during development (Teotia and Lamb, 2011). *HvNAC005* (Christiansen *et al.*, 2011) and *ANAC029/AtNAP*, the orthologous gene in *Arabidopsis* (Guo *et al.*, 2004a), were already described as upregulated during senescence. *HvNAC005* was furthermore shown to be markedly upregulated by ABA treatment (Christiansen *et al.*, 2011).

The hybridization of the microarray identified *HvWRKY12* as the only gene encoding a WRKY factor upregulated during senescence. Its transcript level increased during development in both SN (SN2 vs. SN1) and HN (HN3 vs. HN1) leaves (Table 1A). This result was also confirmed by qRT-PCR using samples collected in the second year (Fig. 3B). Interestingly, the *HvWRKY12* gene was not upregulated during senescence induced by carbohydrate accumulation as a result of steam girdling (Parrot *et al.*, 2007). *AtWRKY75*, its orthologous gene in *Arabidopsis* (Mangelsen *et al.*, 2008), was reported to be upregulated during senescence (Guo *et al.*, 2004b) and in response to pathogens (Chen *et al.*, 2013). *OsWRKY72*, its orthologous gene in rice, could be induced by treatment with ABA (Xie *et al.*, 2005).

In previous studies on senescence-associated gene expression, genes encoding members of the NAC and WRKY transcription factors were shown to have high levels of expression in senescent leaves (Guo *et al.*, 2004a; Buchanan-Wollaston *et al.*, 2005; Balazadeh *et al.*, 2008; Breeze *et al.*, 2011; Fischer, 2012). Here, the two *NAC* genes *HvNAC001* and *HvNAC026* were shown to have highest upregulation in senescent leaves from SN plots in 2009 as analysed by microarrays (Table 1B). This result was similar for *HvNAC026* during qRT-PCR using samples collected in 2010 even if an upregulation in HN plots was also shown (Fig. 3C). For the *HvNAC026* gene an upregulation in senescing barley flag leaves and in the grain at the dough stage was shown (Christiansen *et al.*, 2011). The orthologous gene in *Arabidopsis*, *XND1*, was described to negatively regulate lignocellulose synthesis and programmed cell death in the xylem (Zhao *et al.*, 2007). Orthologues in poplar are involved in xylem formation, leaf growth, and development (Grant *et al.*, 2010). It is likely that during leaf senescence, HvNAC026 acts as a suppressor of cell death processes, thereby allowing efficient nitrogen remobilization. Whether nitrogen remobilization is hampered in transgenic barley plants with reduced level of HvNAC026 remains to be investigated. The *HvNAC001* gene was also upregulated in senescing leaves from SN plots but the transcript level was higher in leaves from HN plots (Fig. 3B). Thus, it is unlikely that HvNAC001 is a regulator of nitrogen remobilization. In other studies on *HvNAC001*, a slight upregulation in senescent leaves and in leaves treated with ABA, but an intensive upregulation in older grains was reported (Christiansen *et al.*, 2011).

### Genes encoding proteins putatively involved in protein degradation during senescence

The second focus of the microarray analyses was on genes encoding proteins involved in protein degradation, in particular proteases. Several proteases have been described to be associated with protein degradation during senescence (Gepstein *et al.*, 2003; Guo *et al.*, 2004a; Schaller, 2004; Buchanan-Wollaston *et al.*, 2005; Parrott *et al.*, 2005, 2007), but for most of them their functional roles in nitrogen remobilization remain to be shown.

HvPAP20 and HvPAP14 are both cysteine peptidases of the papain type. *HvPAP14* was one of the most upregulated genes during development of flag leaves from SN field plots (Table 1A). The gene encodes a HDEL-tailed papain cysteine peptidase. For the orthologous genes in *Ricinus communis* and *Arabidopsis*, an involvement in plant cell death was shown (Gietl and Schmid, 2001; Hierl *et al.*, 2012). Expression patterns of both genes, *HvPAP20* and *HvPAP14*, showed similarities to the expression pattern of the senescence-associated gene *HvSAG12* (Fig. 3A, B), which codes for another papain-type cysteine peptidase. In time course experiments on *Arabidopsis* leaf senescence, *AtSAG12* was upregulated at a later stage of senescence (Breeze *et al.*, 2011). *OsSAG12*-1, the orthologous gene in rice was described as a negative regulator of cell death (Singh *et al.*, 2013). Furthermore, the gene encoding a subtilase-type serine peptidase showed a high upregulation in senescing SN and HN leaves in the microarray experiment (Table 1A). qRT-PCR showed that the expression increased during development only in HN leaves (Fig. 3B). The orthologous gene in *Arabidopsis* codes for a protein identified in the vacuole (Carter, 2004). It was shown to be upregulated in mutants devoid of the microRNA *miR159*, which is involved in the regulation of programmed cell death (Alonso-Peral *et al.*, 2010).

Eight genes encoding cysteine peptidases and two genes encoding serine peptidases were identified as highly upregulated in senescing SN (SN2 vs. SN1) leaves (Table 1B). Two of these genes were selected for further analysis by qRT-PCR with samples collected in the field in 2010. The first gene is *HvPAP20* encoding a cysteine type peptidase, and the second gene is *SCPL51* encoding a serine type protease (Table 1B). qRT-PCR confirmed a higher upregulation during senescence of SN leaves compared with HN leaves for *SCPL51* (Fig. 3C), but not for *HvPAP20* (Fig. 3B). In previous studies, *SCPL51* was shown to be upregulated in germinating barley seeds (Druka *et al.*, 2006; Sreenivasulu *et al.*, 2008), and after anthesis in leaves of a high-grain-protein variety compared with the leaves of a low- grain-protein variety (Jukanti *et al.*, 2008). These results are in accordance with the idea that SCPL51 is involved in nitrogen remobilization, which is likewise important for senescence and germination (Thomas and Smart, 1993; Thomas and Howarth, 2000; Palma *et al.*, 2002; Schaller, 2004). Although *HvPAP20* was upregulated during senescence of SN leaves, its transcript level was higher in the older HN leaves. *HvPAP20* encodes a cathepsin-b-like cysteine protease (Martínez and Diaz, 2008) known to be also upregulated during germination of barley (Druka *et al.*, 2006; Sreenivasulu *et al.*, 2008).

The high upregulation of *ATG7* and *ATG18F* in senescent SN leaves indicates that autophagy is also involved in nitrogen degradation and remobilization in barley flag leaves under field conditions (Table 1B, Fig. 3C). The protein encoded by *ATG7* in barley is involved in the protein conjugation reaction (Mizushima *et al.*, 1998). The autophagy-related gene *ATG18F* was also shown to be upregulated in *Arabidopsis* seedlings exposed to sucrose or nitrogen starvation (Xiong *et al.*, 2005). Besides its role in accomplishment of protein degradation (Guiboileau *et al.*, 2012; Lee *et al.*, 2013), autophagy might suppress cell death by controlling the NPR1-dependent salicylic acid signalling process (Yoshimoto *et al.*, 2009).

In conclusion, the gene expression analyses presented here indicate that the NAC transcription factor HvNAC026, the serine type protease SCPL51, and the autophagy factors APG7 and ATG18F might be major regulators and executors of nitrogen remobilization during barley leaf senescence in the field. Both HvNAC026 and autophagy might be important for remobilization through their negative impact on cell-death processes.

## Supplementary Data

Supplementary data are available at *JXB* online.

Figure S1. Characterization of flag leaves and ears in 2010.

Figure S2. Heat map of the selected and annotated genes identified by the microarray experiment.

Table S1. Microarray expression values.

Table S2. Sequences of primers used for quantitative real-time PCR.

Supplementary Data

## Abbreviations:

HN: high nitrogen
qRT-PCR: quantitative real-time PCR
Rubisco: ribulose-1,5-bisphosphate carboxylase/oxygenase
SD: standard deviation
SN: standard nitrogen
SP2: splicing factor 2.

## References

[CIT0001] Alonso-Peral MM Li J Li Y Allen RS Schnippenkoetter W Ohms S White RG Millar AA 2010 The microRNA159-regulated GAMYB-like genes inhibit growth and promote programmed cell death in *Arabidopsis* . Plant Biology 154, 757–771 10.1104/pp.110.160630 PMC2949021 20699403

[CIT0002] Balazadeh S Riano-Pachon DM Mueller-Roeber B 2008 Transcription factors regulating leaf senescence in *Arabidopsis thaliana* . Plant Biology 10, 63–75 18721312 10.1111/j.1438-8677.2008.00088.x

[CIT0003] Bhalerao R Keskitalo J Sterky F 2003 Gene expression in autumn leaves. Plant Biology 131, 430–442 10.1104/pp.012732 PMC166820 12586868

[CIT0004] Bolstad BM Irizarry RA Astrand M Speed TP 2003 A comparison of normalization methods for high density oligonucleotide array data based on variance and bias. Bioinformatics 19, 185–193 12538238 10.1093/bioinformatics/19.2.185

[CIT0005] Breeze E Harrison E McHattie S 2011 High-resolution temporal profiling of transcripts during Arabidopsis leaf senescence reveals a distinct chronology of processes and regulation. The Plant Cell 23, 873–894 21447789 10.1105/tpc.111.083345 PMC3082270

[CIT0006] Buchanan-Wollaston V Page T Harrison E 2005 Comparative transcriptome analysis reveals significant differences in gene expression and signalling pathways between developmental and dark/starvation-induced senescence in *Arabidopsis* . The Plant Journal 42, 567–585 15860015 10.1111/j.1365-313X.2005.02399.x

[CIT0007] Carter C 2004 The vegetative vacuole proteome of *Arabidopsis thaliana* reveals predicted and unexpected proteins. The Plant Cell Online 16, 3285–3303 10.1105/tpc.104.027078 PMC535874 15539469

[CIT0008] Chen X Liu J Lin G Wang A Wang Z Lu G 2013 Overexpression of AtWRKY28 and AtWRKY75 in *Arabidopsis* enhances resistance to oxalic acid and *Sclerotinia sclerotiorum* . Plant Cell Reports 32, 1589–1599 23749099 10.1007/s00299-013-1469-3

[CIT0009] Christiansen MW Holm PB Gregersen PL 2011 Characterization of barley (*Hordeum vulgare* L.) NAC transcription factors suggests conserved functions compared to both monocots and dicots. BMC Research Notes 4, 302 21851648 10.1186/1756-0500-4-302 PMC3226072

[CIT0010] Diaz C Purdy S Christ A Morot-Gaudry J-F Wingler A Masclaux-Daubresse C 2005 Characterization of markers to determine the extent and variability of leaf senescence in *Arabidopsis*. A metabolic profiling approach. Plant Biology 138, 898–908 10.1104/pp.105.060764 PMC1150406 15923326

[CIT0011] Druka A Muehlbauer GJ Druka I 2006 An atlas of gene expression from seed to seed through barley development. Functional & Integrative Genomics 6, 202–211 16547597 10.1007/s10142-006-0025-4

[CIT0012] Dvinge H Bertone P 2009 HTqPCR: high-throughput analysis and visualization of quantitative real-time PCR data in R. Bioinformatics 25, 3325–3326 19808880 10.1093/bioinformatics/btp578 PMC2788924

[CIT0013] Fischer AM 2012 The complex regulation of senescence. Critical Reviews in Plant Sciences 31, 124–147

[CIT0014] Gepstein S Sabehi G Carp M-J Hajouj T Nesher MFO Yariv I Dor C Bassani M 2003 Large-scale identification of leaf senescence-associated genes. The Plant Journal 36, 629–642 14617064 10.1046/j.1365-313x.2003.01908.x

[CIT0015] Gietl C Schmid M 2001 Ricinosomes: an organelle for developmentally regulated programmed cell death in senescing plant tissues. Naturwissenschaften 88, 49–58 11320888 10.1007/s001140000203

[CIT0016] Grant EH Fujino T Beers EP Brunner AM 2010 Characterization of NAC domain transcription factors implicated in control of vascular cell differentiation in *Arabidopsis* and Populus. Planta 232, 337–352 20458494 10.1007/s00425-010-1181-2

[CIT0017] Gregersen PL Holm PB 2006 Transcriptome analysis of senescence in the flag leaf of wheat (*Triticum aestivum* L.). Plant Biotechnology Journal 5, 192–206 17207268 10.1111/j.1467-7652.2006.00232.x

[CIT0018] Gregersen PL Culetic A Boschian L Krupinska K 2013 Plant senescence and crop productivity. Plant Molecular Biology 82, 603–622 23354836 10.1007/s11103-013-0013-8

[CIT0019] Guiboileau A Yoshimoto K Soulay F Bataillé M-P Avice J-C Masclaux-Daubresse C 2012 Autophagy machinery controls nitrogen remobilization at the whole-plant level under both limiting and ample nitrate conditions in *Arabidopsis* . New Phytologist 194, 732–740 22404536 10.1111/j.1469-8137.2012.04084.x

[CIT0020] Guo Y Cai Z Gan S 2004a Transcriptome of *Arabidopsis* leaf senescence. Plant Cell and Environment 27, 521–549

[CIT0021] Guo Z Kan Y Chen X Li D Wang D 2004b Characterization of a rice WRKY gene whose expression is induced upon pathogen attack and mechanical wounding. Acta Botanica Sinica 46, 955–964

[CIT0022] Hierl G Vothknecht U Gietl C 2012 Programmed cell death in *Ricinus* and *Arabidopsis*: the function of KDEL cysteine peptidases in development. Physiologia Plantarum 145, 103–113 22268582 10.1111/j.1399-3054.2012.01580.x

[CIT0023] Hörtensteiner S Feller U 2002 Nitrogen metabolism and remobilization during senescence. Journal of Experimental Botany 53, 927–937 11912235 10.1093/jexbot/53.370.927

[CIT0024] Humbeck K Quast S Krupinska K 1996 Functional and molecular changes in the photosynthetic apparatus during senescence of flag leaves from field-grown barley plants. Plant Cell and Environment 19, 337–344

[CIT0025] Jansson S Thomas H 2008 Senescence: developmental program or timetable? The New Phytologist 179, 575–579 18433431 10.1111/j.1469-8137.2008.02471.x

[CIT0026] Jukanti AK Fischer AM 2008 A high-grain protein content locus on barley (*Hordeum vulgare*) chromosome 6 is associated with increased flag leaf proteolysis and nitrogen remobilization. Physiologia Plantarum 132, 426–439 18333996 10.1111/j.1399-3054.2007.01044.x

[CIT0027] Jukanti AK Heidlebaugh NM Parrott DL Fischer IA McInnerney K Fischer AM 2008 Comparative transcriptome profiling of near-isogenic barley (*Hordeum vulgare*) lines differing in the allelic state of a major grain protein content locus identifies genes with possible roles in leaf senescence and nitrogen reallocation. The New Phytologist 177, 333–349 18028296 10.1111/j.1469-8137.2007.02270.x

[CIT0028] Kjaersgaard T Jensen MK Christiansen MW Gregersen PL Kragelund BB Skriver K 2011 Senescence-associated barley NAC (NAM, ATAF1,2, CUC) transcription factor interacts with radical-induced cell death 1 through a disordered regulatory domain. Journal of Biological Chemistry 286, 35418–35429 21856750 10.1074/jbc.M111.247221 PMC3195629

[CIT0029] Kohl S Hollmann J Blattner FR 2012 A putative role for amino acid permeases in sink-source communication of barley tissues uncovered by RNA-seq. BMC Plant Biology 12, 154 22935196 10.1186/1471-2229-12-154 PMC3495740

[CIT0030] Krupinska K Mulisch M Hollmann J Tokarz K Zschiesche W Kage H Humbeck K Bilger W 2012 An alternative strategy of dismantling of the chloroplasts during leaf senescence observed in a high-yield variety of barley. Physiologia Plantarum 144, 189–200 22098170 10.1111/j.1399-3054.2011.01545.x

[CIT0031] Lee TA Wetering SWV Brusslan JA 2013 Stromal protein degradation is incomplete in *Arabidopsis thaliana* autophagy mutants undergoing natural senescence. BMC Research Notes 6, 1–1 23327451 10.1186/1756-0500-6-17 PMC3724497

[CIT0032] Lohman KN Gan S Amasino J Amasino RM Manorama C 1994 Molecular analysis of natural leaf senescence in *Arabidopsis thaliana* . Physiologia Plantarum 92, 322–328

[CIT0033] Mangelsen E Kilian J Berendzen KW Kolukisaoglu UH Harter K Jansson C Wanke D 2008 Phylogenetic and comparative gene expression analysis of barley (*Hordeum vulgare*) WRKY transcription factor family reveals putatively retained functions between monocots and dicots. BMC Genomics 9, 194 18442363 10.1186/1471-2164-9-194 PMC2390551

[CIT0034] Martínez M Diaz II 2008 The origin and evolution of plant cystatins and their target cysteine proteinases indicate a complex functional relationship. BMC Evolutionary Biology 8, 198 18616807 10.1186/1471-2148-8-198 PMC2474614

[CIT0035] Masclaux C Valadier MH Brugière N Morot-Gaudry JF Hirel B 2000 Characterization of the sink/source transition in tobacco (*Nicotiana tabacum* L.) shoots in relation to nitrogen management and leaf senescence. Planta 211, 510–518 11030550 10.1007/s004250000310

[CIT0036] Mayer KFX Martis M Hedley PE 2011 Unlocking the barley genome by chromosomal and comparative genomics. The Plant Cell Online 23, 1249–1263 10.1105/tpc.110.082537 PMC3101540 21467582

[CIT0037] Mizushima N Noda T Yoshimori T Tanaka Y Ishii T George MD Klionsky DJ Ohsumi M Ohsumi Y 1998 A protein conjugation system essential for autophagy. Nature 395, 395–398 9759731 10.1038/26506

[CIT0038] Palma JM Sandalio LM Javier Corpas F Romero-Puertas MC McCarthy I del Río LA 2002 Plant proteases, protein degradation, and oxidative stress: role of peroxisomes. Plant Physiology and Biochemistry 40, 521–530

[CIT0039] Parrott DL John MM Fischer AM 2010 Analysis of barley (*Hordeum vulgare*) leaf senescence and protease gene expression: a family C1A cysteine protease is specifically induced under conditions characterized by high carbohydrate, but low to moderate nitrogen levels. The New Phytologist 187, 313–331 20456047 10.1111/j.1469-8137.2010.03278.x

[CIT0040] Parrott DL McInnerney K Feller U Fischer AM 2007 Steam-girdling of barley (*Hordeum vulgare*) leaves leads to carbohydrate accumulation and accelerated leaf senescence, facilitating transcriptomic analysis of senescence-associated genes. The New Phytologist 176, 56–69 17803641 10.1111/j.1469-8137.2007.02158.x

[CIT0041] Parrott DL Yang L Shama L Fischer AM 2005 Senescence is accelerated, and several proteases are induced by carbon ‘feast’ conditions in barley (*Hordeum vulgare* L.) leaves. Planta 222, 989–1000 16034594 10.1007/s00425-005-0042-x

[CIT0042] Peoples MB Dalling MJ 1988 The interplay between proteolysis and amino acid metabolism during senescence and nitrogen reallocation. In: Noodén LD Leopold AC, eds. Senescence and Aging in Plants. San Diego: Academic Press, 181–217

[CIT0043] Pötter E Kloppstech K 1993 Effects of light stress on the expression of early light-inducible proteins in barley. European Journal of Biochemistry 214, 779–786 8319687 10.1111/j.1432-1033.1993.tb17980.x

[CIT0044] Rawlings ND Barrett AJ Bateman A 2012 MEROPS: the database of proteolytic enzymes, their substrates and inhibitors. Nucleic Acids Research 40, 343–350 10.1093/nar/gkr987 PMC3245014 22086950

[CIT0045] Ruuska SA Lewis DC Kennedy G Furbank RT Jenkins CLD Tabe LM 2008 Large scale transcriptome analysis of the effects of nitrogen nutrition on accumulation of stem carbohydrate reserves in reproductive stage wheat. Plant Molecular Biology 66, 15–32 17934784 10.1007/s11103-007-9249-5

[CIT0046] Schaller A 2004 A cut above the rest: the regulatory function of plant proteases. Planta 220, 183–197 15517349 10.1007/s00425-004-1407-2

[CIT0047] Schildhauer J Wiedemuth K Humbeck K 2008 Supply of nitrogen can reverse senescence processes and affect expression of genes coding for plastidic glutamine synthetase and lysine-ketoglutarate reductase/saccharopine dehydrogenase. Plant Biology 10, 76–84 18721313 10.1111/j.1438-8677.2008.00075.x

[CIT0048] Schulze W Schulze E-D Stadler J Heilmeier H Stitt M Mooney HA 1994 Growth and reproduction of *Arabidopsis thaliana* in relation to storage of starch and nitrate in the wild-type and in starch-deficient and nitrate-uptake-deficient mutants. Plant Cell and Environment 17, 795–809

[CIT0049] Singh S Giri MK Singh PK Siddiqui A Nandi AK 2013 Down-regulation of OsSAG12-1 results in enhanced senescence and pathogen-induced cell death in transgenic rice plants. Journal of Biosciences 38, 583–592 23938390 10.1007/s12038-013-9334-7

[CIT0050] Smyth GK 2005 Limma: linear models for microarray data. In: Gentleman R Carey VJ Huber W Irizarry RA Dudoit S, eds. Statistics for Biology and Health. Bioinformatics and Computational Biology Solutions Using R and Bioconductor. New York: Springer-Verlag, 397–420

[CIT0051] Sreenivasulu N Usadel B Winter A 2008 Barley grain maturation and germination: metabolic pathway and regulatory network commonalities and differences highlighted by new mapman/pageman profiling tools. Plant Biology 146, 1738–1758 10.1104/pp.107.111781 PMC2287347 18281415

[CIT0052] Stamp P Herzog H 1976 Flag-leaf senescence and grain growth in certain German varieties of spring wheat (*Triticum aestivum* L.). Zeitschrift fuer Pflanzenzuechtung 77, 330–338

[CIT0053] Stoy V 1973 Assimilate synthesis and distribution as components of yield formation in cereals. Angewandte Botanik 47, 17–26

[CIT0054] Teotia S Lamb RS 2011 RCD1 and SRO1 are necessary to maintain meristematic fate in *Arabidopsis thaliana* . Journal of Experimental Botany 62, 1271–1284 21172813 10.1093/jxb/erq363 PMC3022410

[CIT0055] Thimm OO Bläsing OO Gibon YY Nagel AA Meyer SS Krüger PP Selbig JJ Müller LAL Rhee SYS Stitt MM 2004 MAPMAN: a user-driven tool to display genomics data sets onto diagrams of metabolic pathways and other biological processes. The Plant Journal 37, 914–939 14996223 10.1111/j.1365-313x.2004.02016.x

[CIT0056] Thomas H Howarth CJ 2000 Five ways to stay green. Journal of Experimental Botany 51, 329–337 10938840 10.1093/jexbot/51.suppl_1.329

[CIT0057] Thomas H Smart CM 1993 Crops that stay green. Annals of Applied Biology 123, 193–219

[CIT0058] Usadel B Poree F Nagel A Lohse M Czedik-Eysenberg A Stitt M 2009 A guide to using MapMan to visualize and compare omics data in plants: a case study in the crop species maize. Plant Cell and Environment 32, 1211–1229 10.1111/j.1365-3040.2009.01978.x 19389052

[CIT0059] Xie Z Zhang Z-L Zou X Huang J Ruas P Thompson D Shen QJ 2005 Annotations and functional analyses of the rice *WRKY* gene superfamily reveal positive and negative regulators of abscisic acid signaling in aleurone cells. Plant Physiology 137, 176–189 15618416 10.1104/pp.104.054312 PMC548849

[CIT0060] Xiong Y Contento AL Bassham DC 2005 AtATG18a is required for the formation of autophagosomes during nutrient stress and senescence in *Arabidopsis thaliana* . The Plant Journal 42, 535–546 15860012 10.1111/j.1365-313X.2005.02397.x

[CIT0061] Yoshimoto K Jikumaru Y Kamiya Y Kusano M Consonni C Panstruga R Ohsumi Y Shirasu K 2009 Autophagy negatively regulates cell death by controlling NPR1-dependent salicylic acid signaling during senescence and the innate immune response in *Arabidopsis* . The Plant Cell 21, 2914–2927 19773385 10.1105/tpc.109.068635 PMC2768913

[CIT0062] Zhang H Sreenivasulu N Weschke W 2004 Large-scale analysis of the barley transcriptome based on expressed sequence tags. The Plant Journal 40, 276–290 15447653 10.1111/j.1365-313X.2004.02209.x

[CIT0063] Zhao C Avci U Grant EH Haigler CH Beers EP 2007 XND1, a member of the NAC domain family in *Arabidopsis thaliana*, negatively regulates lignocellulose synthesis and programmed cell death in xylem. The Plant Journal 53, 425–436 18069942 10.1111/j.1365-313X.2007.03350.x

