# Supplementary material for: Identification of predominant genes involved in regulation and execution of senescence-associated nitrogen remobilization in flag leaves of field grown barley
Source: J Exp Bot. 2014 Apr 3;65(14):3963–73. doi: 10.1093/jxb/eru094 (PMC4106439; doi:10.1093/jxb/eru094)
Supplement: Supplementary Data [file supp_65_14_3963__index.html]

Identification of predominant genes involved in regulation and execution of senescence-associated nitrogen remobilization in flag leaves of field grown barley — Identification of predominant genes involved in regulation and execution of senescence-associated nitrogen remobilization in flag leaves of field grown barley — Supplementary Data 

# Identification of predominant genes involved in regulation and execution of senescence-associated nitrogen remobilization in flag leaves of field grown barley

## Supplementary Data

Data files

**Files in this Data Supplement:**

- Supplementary Data - Supplementary Data
- Supplementary Data - Supplementary Data
- Supplementary Data - Supplementary Data
