# Supplementary material for: Identification of predominant genes involved in regulation and execution of senescence-associated nitrogen remobilization in flag leaves of field grown barley
Source: J Exp Bot. 2014 Apr 3;65(14):3963–73. doi: 10.1093/jxb/eru094 (PMC4106439; doi:10.1093/jxb/eru094)
Supplement: Supplementary Data [file supp_eru094_jexbot117788_file001.pdf]

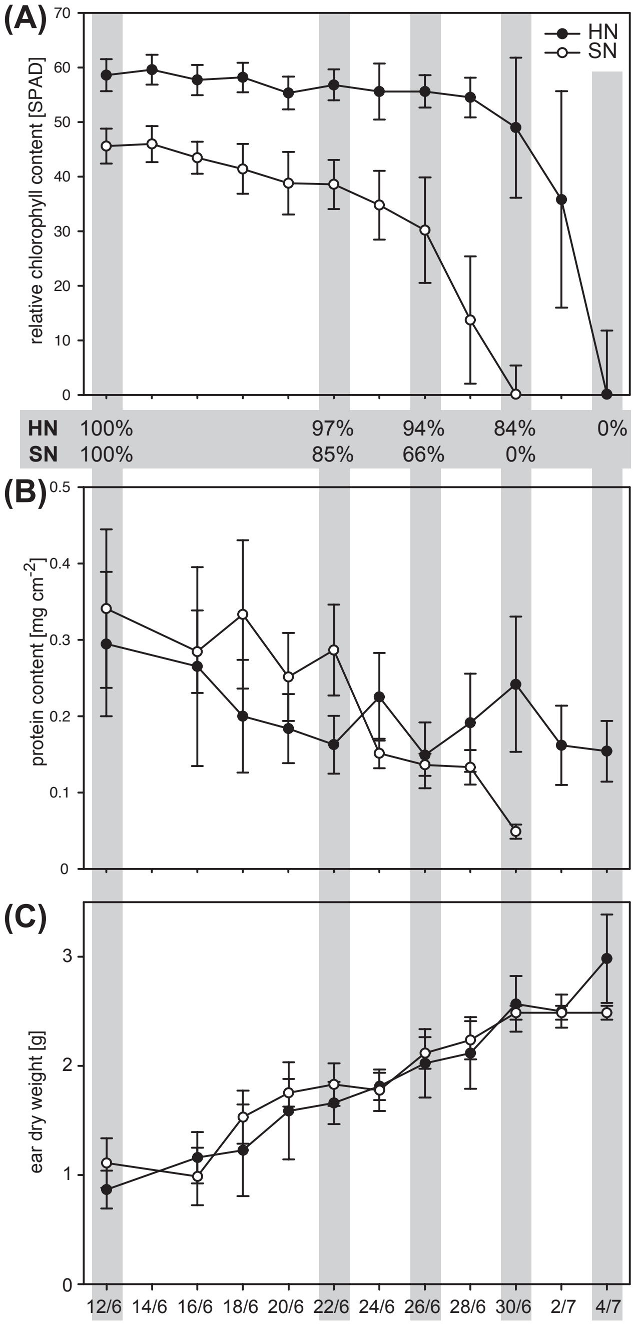

Supplementary Fig. S1. Characterisation of flag leaves and ears collected from barley plants grown in field plots supplied with standard (SN) or high nitrogen (HN) in the period from 12 June (12 days after flowering of SN and HN plants) until 4 July 2010. Samples used for qRT-PCR analyses are highlighted in grey. (A) Relative chlorophyll content. Error bars indicate SD, n = 100. (B) Protein content of leaves. Error bars indicate SD, n = 20. (C) Dry weight of the ears. Error bars indicate SD, n = 12.

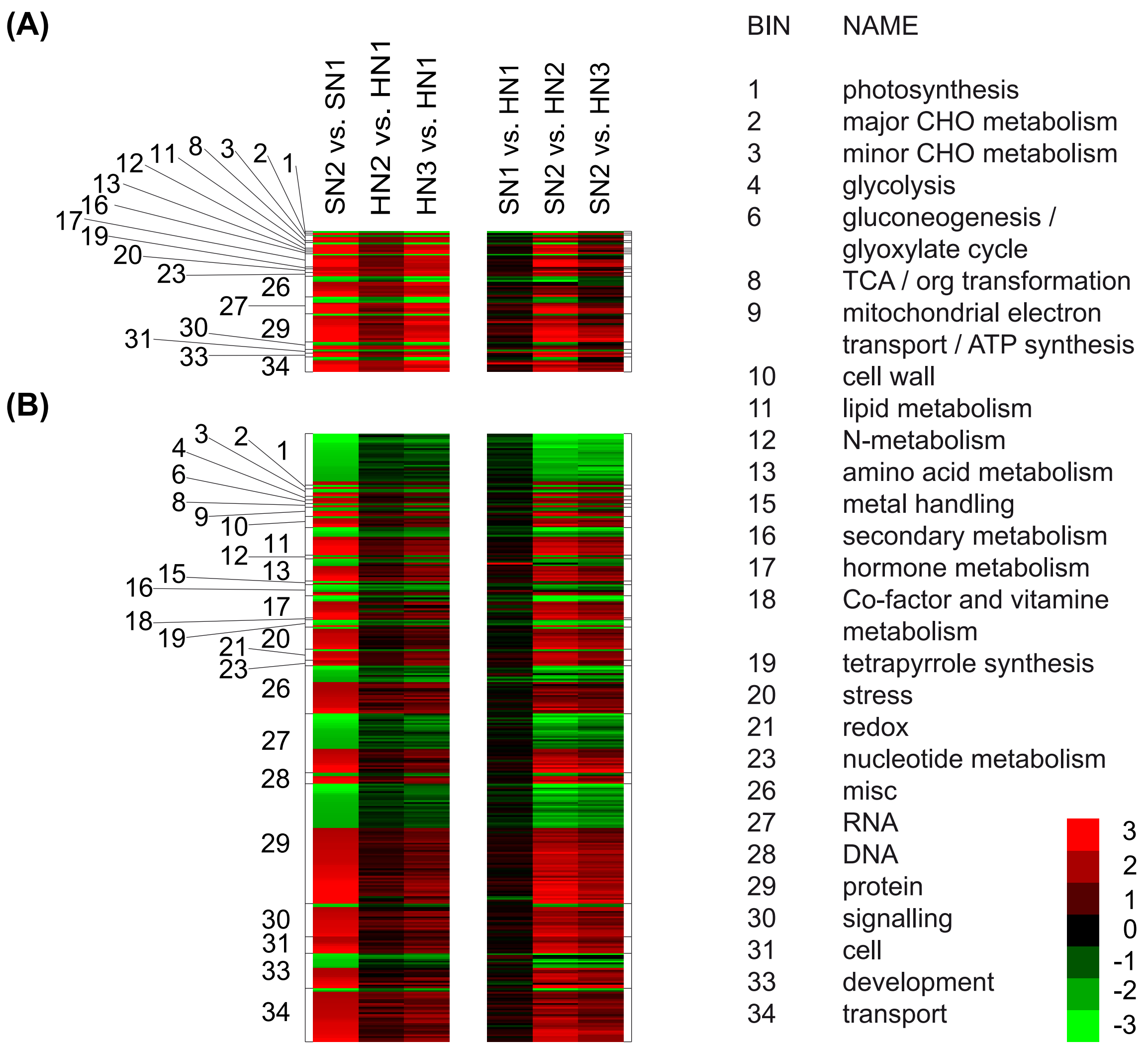

Supplementary Fig. S2. Heat maps of genes differentially expressed between non-senescing and senescing samples from plants grown under standard nitrogen (SN) or high nitrogen (HN) supply after filtering as shown in Figure 2 B. (A) Group of genes min. 2-fold upregulated in the comparison SN2 vs. SN1. (B) Group of genes min. 2-fold upregulated in both comparisons SN2 vs. SN1 and HN3 vs. HN1. Annotations are given as BIN codes (Supplementary Table S1).
